# Using spatial analysis to examine inequalities and temporal trends in food retail accessibility

**DOI:** 10.1017/S1368980024001344

**Published:** 2024-10-24

**Authors:** Cindy Needham, Claudia Strugnell, Liliana Orellana, Steven Allender, Gary Sacks, Miranda R Blake, Ana Horta

**Affiliations:** 1 Deakin University, Global Centre for Preventive Health and Nutrition, Institute for Health Transformation, Geelong 3220, Australia; 2 Deakin University, Institute for Physical Activity and Nutrition, Geelong, Australia; 3 Deakin University, Biostatistics Unit, Faculty of Health, Geelong, Australia; 4 Charles Sturt University, Faculty of Science and Health, Albury-Wodonga, NSW, Australia

**Keywords:** Food retail, Food environment, Health inequalities, Public health nutrition, Supermarket

## Abstract

**Objective:**

In this paper, we examined whether there are inequalities in access to food retail (by type and healthiness) across local government areas (LGA) in Greater Melbourne and by LGA grouped based on their distance from the central business district and Growth Area designation. We also examined whether these inequalities persisted over time.

**Design:**

This is a secondary analysis of a repeated cross-sectional census of food outlets collected at four time points (2008, 2012, 2014 and 2016) across 31 LGA. Using Geographical Information Systems, we present a spatial analysis of food retail environments in Melbourne, Australia, at these four times over eight years.

**Setting:**

Greater Melbourne, Australia.

**Participants:**

31 LGA in Greater Melbourne.

**Results:**

Findings show significant inequalities in access to healthy food retail persisting over time at the LGA level. Residents in lower density urban growth areas had the least access to healthy food retail. Unhealthy food retail was comparatively more accessible, with a temporal trend indicating increased accessibility over time in urban growth areas only.

**Conclusion:**

Accessibility to food outlets, particularly healthy food outlets and supermarkets, in Greater Melbourne is not equal. To identify and address health inequalities associated with rapid urban growth, further understanding of how people interact with the food environment needs to be explored.

In many countries worldwide, a major nutrition transition began in the 1980s which saw changes in the way food was produced, manufactured, transported, priced, promoted and retailed^(1,2)^. This occurred alongside rapid urbanisation, major environmental and social changes^(2)^. As urbanisation continues, the proportion of the population that live in urban areas globally is projected to grow from 55 % in 2018 to 68 % by 2050^(3)^. Sustainable and equitable development of these cities will be essential to support the health of these communities^(1)^. Often, urban growth results in increased urban boundaries allowing low density housing developments to develop on the outskirts of metropolitan areas^(4)^. While this pattern can carry some benefits like increased home affordability and ownership, it also has the potential to magnify inequalities^(4)^. Variations in housing, transportation, basic infrastructure, services and food accessibility between new urban areas and more established urban areas mean the populations needs are met in vastly different ways^(5)^. These inequalities in access to essential resources may influence the health of communities that live within them^(6)^.

In the United States, one study has found that living in lower density residential areas (referred to as urban sprawl) increased the risk of residents being overweight or obese, this risk increased as the measure of urban sprawl increased^(7)^. Despite this evidence, the causal pathway driving this relationship remains unclear^(7)^. Some evidence suggests that inequalities in healthy food accessibility in these more sprawling areas may negatively influence dietary behaviours and overweight and obesity patterns^(8,9)^. The United States Department of Agriculture has sought to address the inequalities in healthy food access by identifying areas, specifically low-income areas, that are food deserts (i.e. have low access to healthy food retail)^(10)^. The United States Department of Agriculture defines low access as areas where 500 people or 33 % of the population live more than one mile (10 miles in rural areas) from a supermarket^(10)^. A clear metric of low access to healthy food is not clearly defined elsewhere, nor is monitoring of food retail accessibility, which together could guide health promotion interventions and assist with public health research and urban planning^(11,12)^.

In Australia, the Victorian Planning Authority released the Precinct Structure Planning Guidelines in 2009 to inform planning for areas experiencing urban expansion which included two recommendations relating to food retail: (1) 80–90 % of households should be within 1 km of a town centre large enough to house a supermarket and (2) local centres include a viable convenience store^(13)^. The purpose of the Victorian Planning Authority recommendations is to ensure equitable access to essential infrastructure (i.e. public transport, public space and housing) as the states’ urban areas continue to grow^(14)^. However, lack of monitoring for population accessibility to town centres, supermarkets or other food retail outlets (hereafter food outlets) means it is not clear whether recommendations are being achieved.

One Australian study examined accessibility to supermarkets in Melbourne, Victoria, in 2013 to see if the recommended 80 % of the population within 1 km of a supermarket was being achieved^(9)^. Using spatial analysis, a road network buffer was created to estimate how many dwellings were within a 1 km distance from a supermarket. Using this measure of accessibility, the study found only 43 % of dwellings in Melbourne were within 1 km of a supermarket. When considering growth areas (designated areas for urban growth with lower residential housing density) alone, only 26 % of dwellings were within 1 km from a supermarket^(9)^. This study did not examine accessibility to other types of outlets that are considered healthy (e.g. greengrocers) or unhealthy (e.g. fast-food) which may also be influential on food purchasing and consumption behaviours. It may be that the presence of supermarkets is in fact an indicator of accessibility to both healthy and unhealthy food outlets (due to a mix of products available at supermarkets) with earlier studies using spatial analysis tools reporting a high correlation between accessibility to unhealthy and healthy outlets in Perth and Melbourne^(15,16)^.

While earlier studies have highlighted inequities in food retail environments, the dominance of cross-sectional (i.e. studies examining the food environment at only a single time point) is a key gap highlighted in the food environment literature given the known, but understudied, changes in the number, type and location of food outlets over time^(12)^. Examining a broad range of food outlets, two Australian studies have demonstrated how the food retail environment is changing over time, giving insight into how the food environment is rapidly expanding and evolving^(8,17)^. One study examined the density of all available food outlets per 10 000 population in Greater Melbourne (2008–2016) and indicated that inequalities in the food environment exist and continue to persist over time, with people living further from the central business district (CBD) and in areas designated for urban growth having a lower density of all food outlets (excluding fast-food)^(17)^. Another study in Perth examined the food environment around the homes of a sample of adults (*n* 2468) between 2004 and 2011 reporting people living in newly established areas, and areas with lower socioeconomic position had lower access to healthy outlets and greater exposure to unhealthy outlets than those living in established areas^(8)^. This imbalance in exposure to healthy and unhealthy outlets warrants further attention given the evidence suggests a higher ratio of unhealthy outlets compared to healthy outlets is associated with a higher BMI in both adults^(18)^ and children^(15)^.

More sophisticated methods taking into consideration a broader range of food outlets and the accessibility routes within the geographical areas of interest are needed to understand the rapid changes occurring in food retail environments (food environments) over time. This would provide insight into factors that may be causing inequities in how communities access basic human needs such as food. Methodological reviews of the literature suggest population-level measures of the food environments (e.g. a whole local government area or state), such as that used by the United States Department of Agriculture, would be more useful than individual measures (e.g. measures of the food environment around an individual’s residences or workplace) for future public health research and policy development^(12)^. Population-based measures of the food environment over a long period of time may provide the opportunity for population-level analysis under the assumption that populations are broadly exposed to the same food environments if they are examined comprehensively^(12)^. Spatial analysis techniques provide the capability to measure accessibility to food outlets across large geographical areas^(19)^, providing for a more accurate representation of the food retail environment and how the population might interact with it^(12)^. Better spatial understanding of the temporal change(s) in food accessibility is critical to identify and address health inequalities associated with rapid urban growth^(11,20)^ and provide the evidence needed to inform best practice for health promotion and urban planning^(19)^. No studies have examined accessibility to the broad array of food outlet types (i.e. more than just supermarkets and fast-food) at the population level (i.e. not just around the homes of a small sample of a population) in Australia, or internationally; or monitored how this changes in areas experiencing rapid growth^(11)^.

The Australian state of Victoria’s capital city, Melbourne, is a prime example where rapid population growth in outer suburbs is happening in parallel with higher obesity rates^(21)^. In this paper, we aim to examine population-level food outlet accessibility from home within (a) walking distance (1 km) and (b) a short drive (3·2 km) to food outlets in Greater Melbourne between 2008 and 2016 and differences in accessibility between these 31 local government areas (LGA). We examine whether there are inequalities in access by grouping LGA based on their distance from the CBD and Growth Area designation. We also examined accessibility in each LGA individually to explore whether most of the population (defined as 80 % of the population) have access to supermarkets (and other outlets) within 1 km of home and whether this changed over time.

## Methods

### Design

This is a secondary analysis of a repeated cross-sectional census of food outlets collected at four time points (2008, 2012, 2014 and 2016) across 31 Melbourne LGA. We chose this eight-year period as during this time rapid population and urban growth was occurring in some LGA^(21)^. In addition, these years align with the years when the Victorian Population Health Surveys collected population health data at the LGA level^(21)^ allowing for future analysis exploring the relationship between population access to food retail at the LGA level and health outcomes over time. The lead author’s institution granted ethics exemption for the present study.

### Food outlets census data

Full details on the data collection process are described elsewhere^(17)^. Briefly, hard copies of residential and business directories (White Pages and Yellow Pages, respectively) for 2008, 2012, 2014 and 2016 were used to identify all food outlets listed in the thirty-one Melbourne LGA using food-related directory classifications and keyword searches. For pragmatic reasons, data extraction excluded a limited number of retailers where the primary product for sale was not food (e.g. liquor stores and pharmacies) or where the business listings were inconsistent across the study years^(17)^. Consistent with prior research^(22)^, food outlets in the CBD (postcode 3000) were excluded due to it being the main business and commercial precinct of Greater Melbourne, with food outlets primarily catering for visitors (e.g. employees and tourists) rather than residents.

Virtual ground truthing was undertaken in May 2019 and involved searching Google and Google Street View (for food outlets at the specified addresses) and food outlet websites for store front and internal photos, as well as details of food offerings (including menus)^(17)^. Google Street View and images posted on Google are time stamped and were therefore useful in retrospectively virtually ground truthing the dataset.

Food outlet name, address and Yellow Pages classification (if available) were extracted. The address was classified by LGA using the Victorian *Electorates by Locality, Postcode and Electorates* dataset^(23)^. The geographic coordinates (i.e. latitude and longitude) of the address were generated using the Google Sheets Geocoder Tool^(24)^ and projected using the Geocentric Datum of Australia 1994 coordinate system in ArcMap 10.5.1. *Open Street Maps Australia – Shops* was used to verify the location of a sample of food outlets (*n* 136, 1 %); 100 % were in the correct location.

### Food outlet type and healthiness classifications

Classifications of food outlet type and healthiness were based on a food environment scoring system for food outlet types in Australian residential communities^(25)^. Australian public health and nutrition experts participated in a modified Delphi study, which resulted in this healthiness rating including twenty-four food outlet types on a scale anchored with –10 (very unhealthy) and +10 (very healthy). Of the twenty-four food outlet types, we dropped eight outlet types that were not included in the original dataset (e.g. ‘pharmacy’ and ‘liquor-selling shop’) and added ‘salad bar/sushi bar’ (healthiness score +5). Online supplementary material, Supplemental Table 1, presents food outlet types and descriptions.

The seventeen food outlet types were collapsed into (a) seven groups (discretionary foods, eating out, fast-food, fresh produce, small goods, supermarkets and takeaways) based on commonalities in food offering definitions, and (b) three groups based on healthiness ratings (healthy, less healthy and unhealthy). In summary, ten different measures (three grouped by healthiness score and seven grouped by type) of the food environment were defined (see online supplementary material, Supplemental Table 2).

### Local government area classification

**Fig. 1:**
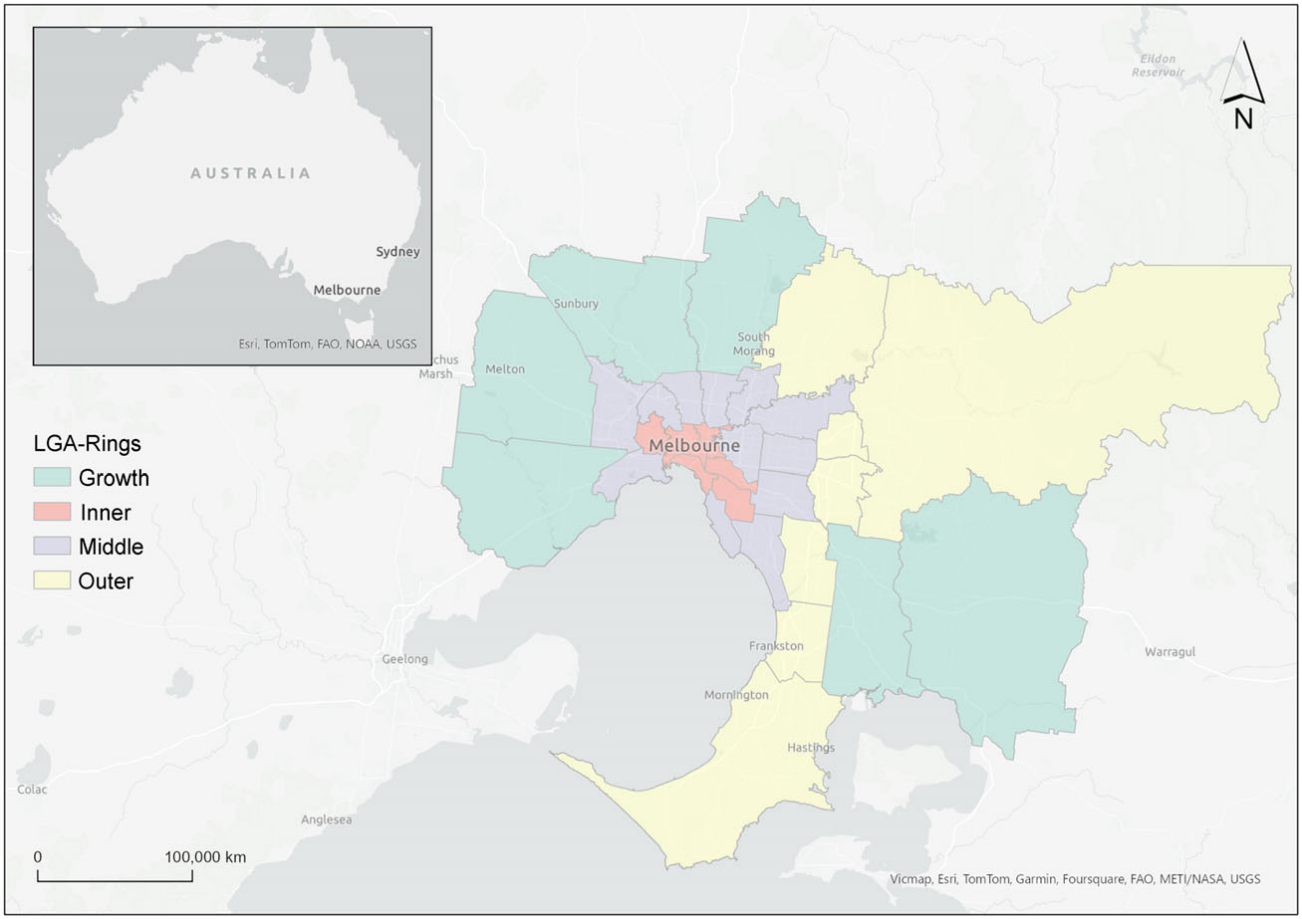
Greater Melbourne local government areas classified by proximity to the Melbourne Central Business District and designated Growth Areas. *Food outlets located in the area of the Central Business District (6·5 km^2^) within the City of Melbourne was excluded from this study as these areas are primarily for commercial and business use rather than residential

LGA were stratified into four routinely used groupings^(6,9)^ cross-referenced with the Victorian Government plan^(26)^ according to their proximity to the Melbourne CBD (Inner ring, *n* 6, Middle ring, *n* 12; or Outer ring, *n* 7) or whether they were designated Growth areas (*n* 6). These groupings are referred to as LGA-Ring (Fig. 1, see online supplementary material, Supplemental Table 3).

### Measures of population access to food outlets within local government areas

For each LGA, we calculated the proportion of the population located within 1 km and 3·2 km of food outlets classified by healthiness score (three levels) and type (seven levels). We first defined 1 km and 3·2 km allocation areas for each food outlet by type and healthiness and then calculated the proportion of population within allocation areas within each LGA using population estimates at the ‘Mesh Block’ (MB) level. The buffer distances of 1 km and 3·2 km were selected based on earlier evidence and recommendations^(9,13,18)^.

### Allocation areas

Allocation areas (i.e. road network buffers), are commonly used to quantify food access and comprise all accessible paths within a specific distance or travel time from a given location using an existing network (e.g. a transport network)^(19,27–29)^. Travel impedance, which refers to the distance or time to travel from the point location to any other location within the allocation range, was used to determine the accessibility within the network. Road network buffers were calculated using the Network Analyst toolset available with the software ArcGIS Pro^(30)^ which applies the Dijkstra algorithm to find the shortest path in a vector-based and topologically connected network^(31)^. The algorithm returns the subset of connected links within a specified range of food outlets and the polygon delimiting the network area covered. The subset of connected links is determined by calculating the cost of traversing a link using physical length as a measure of the cost.

**Fig. 2:**
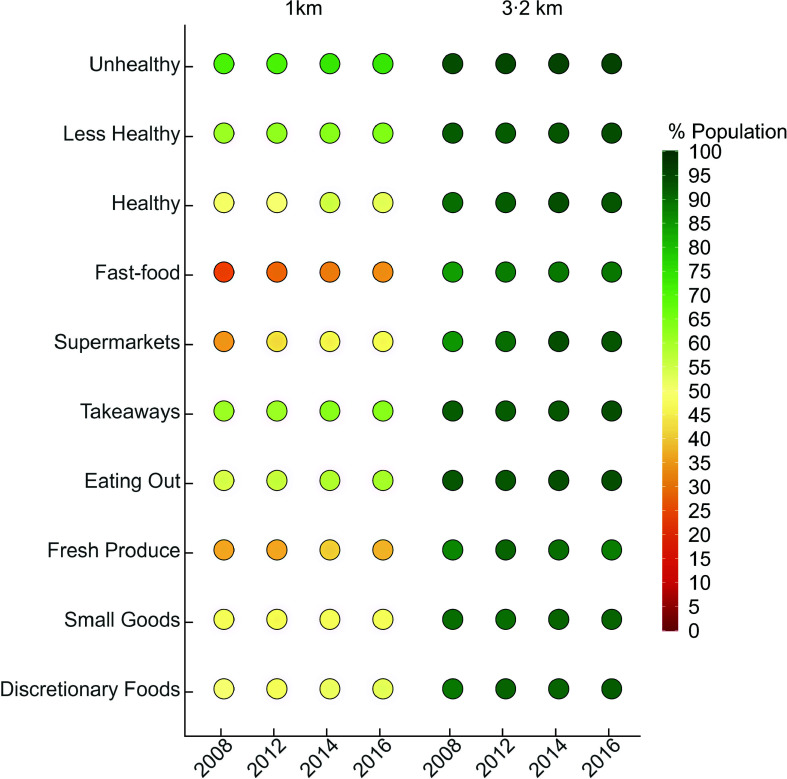
Greater Melbourne allocation areas around Supermarkets in 2016 indicating the proportion of the population within 1 km and 3·2 km of a Supermarket. *Areas located within the allocation areas have access within the defined distance

To create the network dataset needed to generate the allocation areas, we used the Vicmap Transport digital road network which provides ‘an accurate representation of the Transport network across Victoria, at a capture scale ranging from 1:2500 to 1:25 000’^(32)^. The network dataset was created for an area larger than the study area to ensure allocation accuracy. Other details on how network analysis was set are provided in the GEO-Fern Checklist (see online supplementary material, Supplemental Table 4)^(29)^. A total of eighty layers were created representing allocation areas for combinations of the two levels of distance (1 km and 3·2 km), ten food outlet classifications and four study years (Fig. 2).

### Population within allocation areas

To calculate the population within 1 km and 3·2 km of food outlets (based on the allocation areas), we used the Australian Bureau of Statistics Census data for MB population available for 2011 and 2016. MB are the smallest geographical areas for which census data are available. Most residential MB in urban areas contain between 30 and 60 dwellings^(33)^. The population within each MB for 2008, 2012, 2014 and 2016 was calculated based on the rate of change in the Estimated Resident Population (2001–2016) provided by the Australian Bureau of Statistics^(34)^.

The proportion of population within each LGA served by the 1 km and 3·2 km allocation areas was calculated using an areal weighting approach^(35)^ assuming a uniform distribution of the population. Firstly, to identify the MB within the allocation areas, the two layers were spatially overlayed. The population associated with the allocation areas was then calculated as the sum of the MB population. When a MB was only partially included, the ratio between its total area and the partial area was applied to the total MB population. Finally, these numbers were aggregated for each LGA using a spatial join operation to identify the associated MB.

### Statistical analysis

The overall proportion of people with access to food outlets across Greater Melbourne is reported. Linear mixed models were fitted to estimate the mean proportion of the population with access to each food outlet classification within 1 km or 3·2 km by LGA-Ring and year. Twenty outcomes were considered which were defined by accessibility (within 1 km and 3·2 km) for each healthiness/type of food outlet. For each outcome, the model included year (2008, 2012, 2014, 2016), LGA-Ring (Inner, Middle, Outer, Growth Area) and the interaction LGA-Ring by year as fixed effects and LGA as random effects. Sidak adjusted pairwise comparisons are reported: (a) within LGA-Rings between years where the interaction was significant (*P* < 0·05); and (b) between LGA-Rings and/or between years where the interaction was NS. Accessibility to each food outlet measures (by type and healthiness) for each LGA was reported to ascertain whether individual LGA were achieving the recommended standard for 80 % of the population to be within 1 km of a town centre large enough to house a supermarket and mix of retail opportunities.

## Results

### Spatial accessibility in Greater Melbourne 2008–2016

**Fig. 3:**
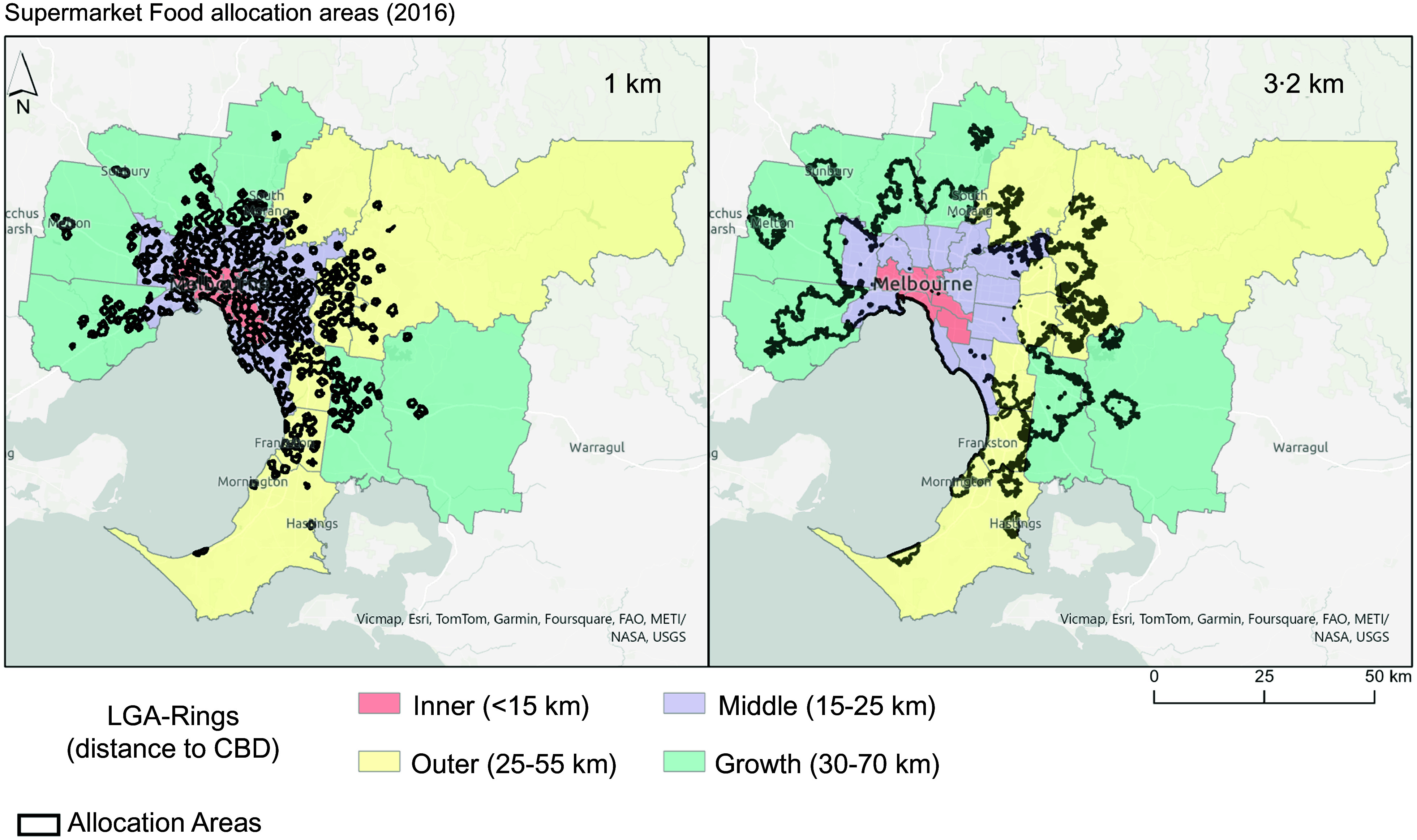
Proportion of the population with access to different food retail outlet types within 1 km and 3·2 km network buffers in Greater Melbourne between 2008–2016. *Areas located within the allocation areas have access within the defined distance

When access to food retail outlets within Greater Melbourne as a whole (i.e. all LGA/LGA-Rings) was examined, the proportion of people with access within 1 km slightly increased over time for Healthy (4·2 percentage points (pp)), Less healthy (2·5pp), and Unhealthy (3·3pp) outlets (Fig. 3, see online supplementary material, Supplemental Table 5) between 2008 and 2016. Across the study period, close to three quarters of the population had access within 1 km to unhealthy outlets, almost two-thirds to less healthy outlets and about half to healthy outlets. When considering food outlet type, the largest increase over time in access within 1 km was to fast-foods outlets (10·5pp) and supermarkets (11·0pp). Access to other types of food outlets changed negligibly.

Most of the population had access to food outlets within 3·2 km, and this proportion increased over time. The largest increase in access within 3·2 km was for supermarkets (increasing from 85 % in 2008 to 92·8 % in 2016) and fast-food outlets (84·5 % in 2008 to 89·4 % in 2016). Figure 3 represents the proportion of the population in Greater Melbourne as a whole with accessibility to food outlets within 1 km and 3·2 km, and online supplementary material, Supplemental Table 5, presents the descriptive statistics.

### Accessibility classified by type and healthiness within 1 km from home, by LGA-Ring and by year

#### Healthiness classification

Across each of the three measures, the proportion of people with access to any type of food outlet within 1 km decreased progressively from the Inner, Middle, Outer to Growth Area LGA-Rings.

The temporal profile of the percentage of people with access to Healthy, Less healthy and Unhealthy food outlets differed across LGA-Rings (Table 1, all interactions *P* < 0·05). The main difference in temporal trends was the positive trend in Growth Areas (i.e. increase in the number of people with access to all food outlets), with smaller changes in other LGA-Rings (see online supplementary material, Supplemental Table 6a).

**Table 1:** Mean proportion of the population within 1 km of food retail outlets within local government areas classified by distance from the central business district and identified growth area between 2008 and 2016

	Local government area ring																*P*-value: model with interaction
	Inner				Middle				Outer				Growth				
Mean proportion (%, 95 % CI) of the population within LGA stratified by distance from the CBD within 1 km of food outlets classified by healthiness																	
Healthy	**88**	**84**	**93**	**88**	**59**	**62**	**66**	**64**	**31**	**34**	**39**	**36**	**19**	**22**	**29**	**28**	**0·0240**
95 % CI	88, 78	84, 75	93, 83	88, 78	59, 52	62, 55	66, 59	64, 57	31, 22	34, 25	39, 30	36, 27	19, 9	22, 13	29, 20	28, 18	
Less Healthy	**97**	**97**	**97**	**98**	**77**	**78**	**79**	**80**	**42**	**44**	**44**	**45**	**25**	**26**	**31**	**33**	**0·0009**
95 % CI	97, 87	97, 87	97, 88	98, 88	77, 70	78, 71	79, 72	80, 73	42, 33	44, 35	44, 35	45, 36	25, 16	26, 16	31, 21	33, 23	
Unhealthy	**97**	**97**	**98**	**98**	**85**	**84**	**87**	**87**	**54**	**54**	**57**	**57**	**39**	**42**	**49**	**51**	**< 0·0001**
95 % CI	97, 86	97, 86	98, 86	98, 86	85, 77	84, 76	87, 79	87, 79	54, 44	54, 43	57, 47	57, 46	39, 27	42, 31	49, 37	51, 40	
Mean proportion (%, 95 % CI) of the population within LGA stratified by distance from the CBD within 1 km of food outlets classified by outlet type																	
Discretionary	**83**	**83**	**85**	**85**	**59**	**54**	**60**	**62**	**35**	**32**	**37**	**38**	**24**	**25**	**29**	**31**	0·1219
95 % CI	73, 93	72, 93	75, 95	74, 95	52, 66	46, 61	52, 67	55, 69	25, 43	22, 40	27, 46	28, 47	13, 34	15, 36	19, 39	20, 40	
Eating out	**95**	**96**	**97**	**97**	**70**	**72**	**74**	**75**	**37**	**40**	**40**	**41**	**20**	**22**	**27**	**29**	**0·0055**
95 % CI	95, 86	96, 86	97, 87	97, 88	70, 63	72, 65	74, 67	75, 69	37, 29	40, 32	40, 32	41, 32	20, 10	22, 12	27, 18	29, 19	
Fast-food	**44**	**56**	**63**	**66**	**27**	**33**	**38**	**40**	**15**	**19**	**20**	**21**	**10**	**14**	**15**	**17**	**< 0·0001**
95 % CI	38, 51	49, 62	57, 70	59, 72	23, 32	29, 38	34, 43	36, 45	9, 21	13, 25	14, 26	16, 27	4, 17	7, 20	9, 22	10, 23	
Fresh produce	71*	64*	72*	68*	48*	48*	52*	51*	22*	23*	25*	23*	12*	15*	16*	14*	**0·0175**
95 % CI	62, 80	55, 73	63, 80	59, 76	41, 54	42, 54	46, 58	45, 57	14, 30	15, 31	17, 33	15, 32	3, 21	7, 24	8, 25	6, 23	
Small goods	**84**	**83**	**84**	**84**	**58**	**57**	**58**	**58**	**29**	**29**	**30**	**29**	**20**	**20**	**21**	**22**	0·9140
95 % CI	75, 93	74, 92	76, 93	75, 93	52, 64	51, 63	52, 64	52, 64	20, 37	21, 38	22, 38	21, 37	11, 29	11, 28	13, 30	13, 31	
Supermarkets	**68**	**75**	**79**	**79**	**41**	**52**	**54**	**55**	**22**	**26**	**29**	**28**	**12**	**18**	**25**	**24**	**0·0333**
95 % CI	68, 58	75, 65	79, 70	79, 70	41, 35	52, 46	54, 47	55, 48	22, 13	26, 17	29, 20	28, 20	12, 3	18, 8	25, 15	24, 15	
Takeaways	90**	93**	95**	96**	76**	76**	78**	79**	43**	42**	44**	45**	30**	30**	34**	37**	0·4691
95 % CI	78, 101	82, 105	84, 107	85, 108	68, 84	68, 85	70, 86	71, 87	33, 54	32, 53	34, 55	34, 55	18, 41	19, 42	22, 45	25, 48	

CBD: Central Business District; LGA: local government areas

Mean estimates and 95 % CI obtained under linear mixed including years (2008, 2012, 2014 and 2016), LGA-Ring (Inner, Middle, Outer, Growth Area) and the interaction LGA-Ring by year as fixed effects and LGA as random effects; significance of interaction and test for main effects from the model is also reported. Significance for model Year effect: Underlined < 0·0001, Underlined* < 0·001, Underlined** < 0·05. Significance for model LGA effect: Bold < 0·0001.

##### Healthy food outlets

Across all years, there were significant differences between LGA-Rings in access to Healthy food outlets within 1 km of home, except for Outer *v*. Growth LGA (Table 1, see online supplementary material, Supplemental Table 6a). The largest contrast between LGA-Rings was the proportion of people with access to Healthy food outlets between the Inner (88·6 %) and Growth Areas (19·4 %) in 2008 (Table 1). Disparity in access decreased over time due to increased access to healthy food outlets in Growth areas. Access increased between 2008 and 2016 for the Middle Ring (5pp), Outer Ring (4pp) and Growth Areas (9pp) but not for Inner Ring (see online supplementary material, Supplemental Table 6a).

##### Less healthy food outlets

Growth and Outer had similar levels of access for all years, as did the Inner and Middle for all years excluding 2008. Inner and Middle had significantly higher access compared to Outer and Growth Rings for all years. The discrepancy in access between Inner and Growth Areas decreased between 2008 (–71 %) to 2016 (–64 %) reflective of increased accessibility in Growth Areas between 2008 and 2016 (Table 1, see online supplementary material, Supplemental Table 6a).

##### Unhealthy food outlets

Unhealthy outlets had the highest accessibility within 1 km compared to all other food outlet types (Table 1). Across all years, Growth and Outer had similar levels of access to unhealthy food outlets, as did Inner and Middle. Inner and Middle had significantly higher access compared to Outer and Growth for all years. Accessibility to Unhealthy outlets only significantly increased with time in the Growth Ring. By 2016, difference in access had reduced between Inner and Growth reflecting an 11pp increase in unhealthy food access in Growth Areas as Inner remained relatively constant (see online supplementary material, Supplemental Table 6a).

#### Food outlet type classification

Access within 1 km to any food outlet type decreased progressively from Inner, Middle, Outer to Growth Areas. Temporal trends in accessibility showed increased accessibility over time to Fast-food, Eating Out and Supermarkets, with a small but significant increase in access to Fresh Produce in the Inner and Middle Rings only (Table 1, see online supplementary material, Supplemental Table 6b).

Across all years, Growth and Outer had similar levels of access to Eating Out, Fast-food, Fresh Produce and Supermarkets (i.e. interaction was significant), while access was significantly different between all other LGA-Rings. There was a significant increase between 2008 and 2016 in access to Eating Out outlets for Middle Ring (5pp) and Growth Areas (9pp). Fast-Food access increased in the same period in all LGA-Rings but at a different rate for the Inner (21pp) and Middle (13pp), with the Outer (6pp) and Growth Areas (6pp) increasing at a similar rate (Table 1, see online supplementary material, Supplemental Table 6b). Access to Fresh Produce only increased in the Inner between 2008 and 2014 (by up to 8pp) and in the Middle between the same period (4pp). Supermarket access increased across all LGA-Rings between 2008 and 2016; Inner (11pp), Middle (13pp), Outer (7pp) and Growth (12pp).

The mean proportion of the population with access to Discretionary, Small Goods and Takeaway outlets within 1 km was significantly different between LGA-Rings except for Outer *v*. Growth and when Middle was compared to Inner for Takeaway (see online supplementary material, Supplemental Table 7).

Between 2008 and 2016, there was a significant overall increase in access to Takeaways (4pp) but no significant change for Discretionary or Small Goods (see online supplementary material, Supplemental Table 7).

### Proportion of local government areas that met the state government mandate for supermarket access

Over the study period, there was an 11pp increase in the proportion of the population with access to a Supermarket within 1 km, with close to 46 % of the population having access in 2016 (Fig. 3).

Only two of thirty-one LGA had more than 80 % of the population within 1 km of a Supermarket in 2008, and only three in 2016 and all were LGA in the Inner Ring. Proportion of the population with access to Supermarkets over the study period did not change for four LGA (two Inner and two Outer, < 2pp), whereas two LGA experienced an increase of 24–25 %pp (one Inner and one Middle LGA). Despite an increase over time, the remaining LGA-Rings in 2016 were far from reaching the mandate (Fig. 2 demonstrates allocation areas around Supermarkets in 2016).

Online supplementary material, Supplemental Table 8, presents sum of local governments where most of (≥ 80 %) the population live within 1 km of food retail outlet types classified by healthiness and food outlet type.

### Accessibility within 3·2 km from home by LGA-Ring between 2008 and 2016

#### Healthiness classification

When compared by LGA-Ring (across years between levels), the trend in accessibility to food outlets classified by healthiness within 3·2 km service areas was significantly different across LGA-Rings (Table 2, see online supplementary material, Supplemental Table 7 and 11). The Inner and Middle LGA-Ring had 100 % of the population within 3·2 km of all food retail and experienced no change. Over time access increased significantly in the Growth Area Ring to Healthy, Less Healthy and Unhealthy outlets (see online supplementary material, Supplemental Tables 10 and 11).

**Table 2:** Mean proportion of the population within local government areas classified by distance from the central business district and identified growth area within 3·2 km of food retail outlets between 2008 and 2016

Local Governments classified by Distance from CBD and identified Growth Area																			
	Inner				Middle				Outer				Growth				Model		
	2008	2012	2014	2016	2008	2012	2014	2016	2008	2012	2014	2016	2008	2012	2014	2016	Year effect	LGA-Ring effect	Interaction
Measure % 3·2 km	Proportion of the population within 3·2 km (%, 95 % CI)																	*P*-value	
Classified by healthiness																			
Healthy	100	100	100	100	100	100	100	100	80	80	86	84	68	82	87	85	1·0000	**< 0·0001**	**< 0·0001**
95 % CI	89, 111	89, 111	89, 111	89, 111	92, 107	92, 107	92, 108	92, 108	69, 90	70, 90	75, 96	74, 94	57, 79	71, 93	76, 98	74, 96			
Less Healthy	100	100	100	100	100	100	100	100	85	82	83	85	77	80	85	86	1·0000	**0·0022**	**0·0001**
95 % CI	89, 111	89, 111	89, 111	89, 111	92, 108	92, 108	92, 108	92, 108	74, 95	72, 93	73, 94	75, 95	66, 88	69, 91	74, 96	75, 98			
Unhealthy	100	100	100	100	100	100	100	100	87	87	87	88	85	88	91	92	1·0000	**0·0037**	**0·0002**
95 % CI	92, 108	92, 108	92, 108	92, 108	94, 105	94, 106	94, 106	94, 106	80, 95	79, 95	80, 95	80, 95	76, 93	80, 96	83, 99	84, 100			
Classified by food outlet type																			
Discretionary	100	100	100	100	99	99	99	99	81	79	80	80	67	75	81	82	1·0000	**0·0004**	**< 0·0001**
95 % CI	86, 114	86, 114	86, 114	86, 114	89, 109	89, 109	89, 109	90, 109	68, 94	67, 92	67, 92	67, 93	53, 80	61, 88	67, 94	69, 96			
Eating out	100	100	100	100	100	100	100	100	84	84	85	86	79	81	85	86	1·0000	**0·0005**	**0·0014**
95 % CI	90, 110	90, 110	90, 110	90, 110	93, 107	93, 107	93, 107	93, 107	75, 93	75, 93	76, 93	77, 95	70, 89	72, 91	76, 95	77, 96			
Fast-food	100	100	100	100	93	96	98	97	69	74	75	75	65	73	76	79	0·9997	**< 0·0001**	**0·0305**
95 % CI	88, 112	88, 112	88, 112	88, 112	85, 102	87, 104	89, 106	89, 106	58, 80	63, 85	64, 86	64, 87	53, 77	61, 85	64, 88	67, 91			
Fresh produce	100	100	100	100	97	100	100	100	78	78	77	77	63	79	78	72	1·0000	**< 0·0001**	**0·0001**
95 % CI	87, 113	87, 113	87, 113	87, 113	88, 106	90, 109	90, 109	90, 109	66, 90	66, 90	65, 89	65, 89	50, 77	65, 92	65, 91	59, 86			
Small goods	100	100	100	100	100	100	100	100	80	79	80	80	73	75	81	82	1·0000	**0·0007**	**< 0·0001**
95 % CI	87, 113	87, 113	87, 113	87, 113	91, 109	91, 109	91, 109	91, 109	69, 92	68, 91	68, 91	68, 91	60, 85	63, 88	68, 93	69, 94			
Supermarkets	100	100	100	100	97	100	100	100	78	79	84	84	55	74	86	83	1·0000	**< 0·0001**	**< 0·0001**
95 % CI	89, 111	89, 111	89, 111	89, 111	89, 105	92, 107	92, 107	92, 108	68, 88	69, 89	74, 95	74, 94	44, 66	63, 85	75, 97	72, 94			
Takeaways	100	100	100	100	100	100	100	100	82	82	82	82	80	80	87	88	1·0000	**0·0037**	**< 0·0001**
95 % CI	89, 111	89, 111	89, 111	89, 111	92, 107	92, 108	92, 108	92, 108	71, 92	72, 92	72, 93	72, 93	69, 92	69, 91	76, 98	77, 99			

CBD: Central Business District; LGA: local government areas

Mean estimates and 95 % CI obtained under linear mixed including year (2008, 2012, 2014 and 2016), LGA-Ring (Inner, Middle, Outer, Growth Area) and the interaction LGA-Ring by year as fixed effects and LGA as random effects; significance of interaction and test for main effects from the model is also reported.

Bold: *P* ≤ 0·05.

#### Food outlet type classification

The Inner and Middle LGA-Ring had the majority of the population within 3·2 km of all food outlets classified by type and experienced no change (Table 2). Comparatively, the Outer and Growth Area LGA had significantly lower access to all food retail outlet types which diminished over time. A significant increase in access to Discretionary, Fresh Produce, Eating Out, Small Goods, Supermarkets and Takeaways was observed only in the Growth Area LGA with the largest increase in access to Supermarkets (30 %pp between 2008 and 2014). Fast-food was the only food outlet type which had no significant change in access across LGA-Rings (see online supplementary material, Supplemental Table 12 and 13).

## Discussion

This study demonstrated significant inequalities in access to food outlets across LGA within one major city experiencing rapid growth in Australia^(6)^. Despite increasing accessibility over time, healthy food outlets such as Fresh Produce (e.g. greengrocers) as well as Supermarkets remain least accessible in the Growth Area LGA of Melbourne. Our results show accessibility decreased as distance from the CBD increased. For example, almost 90 % of those living closest to the CBD (Inner LGA) had access to a Healthy food outlet within 1 km, compared to 25 % in Growth LGA. In contrast, in 2016 half the population in Growth Areas had access to an Unhealthy Outlet within 1 km, making Unhealthy food outlets more accessible than Healthy outlets within an estimated ‘reasonable’ walking distance. Growth Areas were the only LGA-Ring to experience significant growth in the proportion of the population with access to Unhealthy outlets within 1 km and 3·2 km between 2008 and 2016.

Our results are consistent with results from an earlier study examining the changing food environment (2004–2011) in Perth, Western Australia^(8)^. Together the evidence shows that across two major cities in Australia disparity in spatial access to food retail, particularly healthy food retail, is prominent^(8)^. More recent studies examining how the food retail environment is changing over time in high-income countries are scarce and likely a result of the challenges to obtaining food retail data and the time-consuming nature of food retail outlet classification, particularly for large urban geographical areas^(36)^. However, observed changes in developing countries such as China^(37)^ and more recently Mexico^(38)^ suggest a similar trend of increasing access to unhealthy food retail outlets (i.e. fast-food franchise and convenience stores) over healthier food retail outlets (e.g. grocery stores) alongside urbanisation.

Raising awareness of these disparities so as to influence future policy and planning interventions is needed given the evidence that suggests geographic (and economic) availability of food outlets has been shown to drive healthiness of diets^(39,40)^. Further evidence suggests that the relationship between the food environment and obesity is likely driven by its influence on dietary behaviours. This is demonstrated in a growing body of evidence showing a positive relationship that exists between increased availability of fruit and vegetables and increased consumption^(41)^. Further, adults living farthest away from a supermarket in Cambridge, United Kingdom, were shown to have a 15 % lower odds of having a healthy diet (using the DASH-accordant diet) compared to those living closer^(40)^. Additionally in both Canada and Australia evidence demonstrates an inverse relationship between access to healthy food outlets and supermarkets and the risk of obesity in both children and adults^(15,42)^.

Our findings support the need for further research to explore whether inequalities in healthy food outlet access are contributing to the disproportionate prevalence of people with overweight and obesity in some LGA compared to others^(21)^. Currently, Victorian planning laws do not require food outlet accessibility to be monitored or the healthiness of food outlets to be considered in urban planning. Findings support the need for food outlet accessibility to be actively considered as part of health promotion efforts to improve diets. This could be achieved by providing more progressive planning powers to local governments, following suit with powers provided to local governments in England which ensure planning for healthy weight environments is a key consideration of planning policy^(43)^.

Population measures of food outlet accessibility will be critical to evaluate whether policies aimed at increasing the healthiness of food environments are achieving the desired outcomes (e.g. increasing healthy food outlet access) and will provide the opportunity for ecological studies examining food environments and their influence on population health^(12)^. Given vastly different levels of accessibility across LGA demonstrated in this study, population analysis at the LGA level may be an appropriate scale for future ecological studies. Examining the food environment and its influence on the population at the LGA level would advance the evidence, by removing challenges associated with individual level food environment research, findings from which cannot be applied to the broader population^(12,44)^.

No recent studies are available to demonstrate more current trends (i.e. 2017 and beyond) in the evolving food retail environment in Greater Melbourne. However, Australia’s National Science Agency report suggests Australian food and agribusinesses will see an estimated growth of 3·6 % annually of health and wellness foods, and sustainable and premium (i.e. luxury convenience) foods between 2018 and 2030^(45)^. Expected industry growth is thought be partly driven by changing preferences of an ageing consumer population in Australia^(45)^. This includes growing demand for healthy and accessible foods that are minimally processed, ethically and sustainably sourced to support better health and wellbeing^(45,46)^. These domestic demand-side drivers are likely to be reflected in the food retail environment through increased access to healthy food retail opportunities within communities.

### Strengths

This study presented a more holistic approach to examining food environments (i.e. examining the majority of food retailers) than presented in earlier studies focused largely on measures of accessibility to large chain fast-food outlets and supermarkets^(42)^. Further, by taking into consideration the road networks that enable access to food outlets^(17)^ this study provides a more detailed indication of food outlet accessibility using measures of access to the majority of food retail within an entire city as opposed to only a small number of food outlet types around a sample of individual households^(8)^. We demonstrate the dynamic nature of food environments in a city experiencing rapid growth and provide further evidence supporting the need for food environment research to take both spatial and temporal approaches.

### Limitations

This paper examined food outlet accessibility to residential dwellings in Melbourne Victoria. Inferential errors may occur when using routine administrative units (e.g. LGA) to represent an individual’s food retail environment^(47)^. Including pharmacies and liquor-selling stores was not possible in this study; however, it would be beneficial to include these outlets in future research as they are readily accessible in urban areas and retailers of unhealthy food and beverages. Food environments near workplaces and school environments may also influence the foods purchased and consumed and needs to be understood. It would have been valuable to understand if the LGA socioeconomic position played a role in inequalities. However, variation in area-level disadvantage at the LGA level was too low to undertake robust analysis as part of this study. This study did not capture data relating to food delivery opportunities, which have increased over time^(48)^. MB assume that the population is evenly distributed which might not be the case particularly in residential MB in less populated areas. The allocation areas were calculated based on distance in the network and not travel time due to lack of data, thus not accounting for time as an enabling factor of food outlet accessibility. Finally, the data presented in this study were restricted to availability of hard copy business listing which were not available from 2017 onwards and therefore may not represent current food retail accessibility. Nevertheless, the reported findings present the first insight into food retail accessibility patterns in Greater Melbourne over time which will provide a platform for future comparison where data is available.

### Recommendations for future research and policy development

With continued urban growth, longitudinal studies examining the impact of more accessible food environments (e.g. walkable food environments) compared to more sprawling (e.g. most need to drive to access food outlets) food environments on health and health behaviours need to be considered. Research outside metropolitan cities would broaden our understanding of food environments, as would exploring the influence of online food delivery opportunities^(48)^. The extent to which inequalities in food outlet accessibility follow a socioeconomic gradient would also provide valuable insight into the challenges faced by these populations. Research also needs to be undertaken to understand the role Supermarkets play in the provision of healthy and unhealthy food to determine whether they can continue to be used as a proxy for healthy food retail availability.

A review of Australian government food and diet-related policies highlights that several states have made progress towards supporting healthier food environments by incorporating ‘community health and wellbeing’ as a consideration in the planning system^(49)^. However, to our knowledge these policies are yet to be implemented through planning policies and provisions. In England, local government implementation of planning policies to prevent new unhealthy food outlets near schools, where the density of unhealthy outlets or where childhood obesity has surpassed a certain threshold, has been successful in reducing the density and proportion of unhealthy food outlets^(50)^. The development of similar policies in Australia, and monitoring of their effectiveness, may be a promising step towards reducing inequalities and increasing the healthiness of food environments.

### Implications for health promoting policy and practice

There is an established relationship between access to healthy outlets and supermarkets and healthier body composition^(15,42)^. Poor access to food outlets, particularly healthy food outlets, may influence consumption of healthy food and widen inequalities, disadvantaging those with limited car access or public transport options and scarce financial resources^(1)^. To further strengthen the policy position, valuable insights could be learnt from Public Health England who provided local authority public health and planning teams’ powers to promote healthy weight environments^(43)^. Guidance provided indicates six elements by which planners can promote healthy weight environments, one of which is to improve the food environment for both consumption and production of healthier options^(43)^. For guidelines to be impactful requires accountability and enforcement of the planning guidelines, with ongoing monitoring of access and temporal trends. In Australia, food environment researchers will need to collect food environment data from other sources (e.g. local governments, commercial datasets) given the discontinued publication of Yellow pages business listings used in this study. Population measures of accessibility to food outlets are a valuable resource for public health practitioners wishing to address issues such as food insecurity and healthy diet by pinpointing areas of low access; also providing the opportunity to examine whether there is a relationship between the shared environment and measures of population health^(12)^. To address the issues of poor access to healthy food retail may require the implementation of initiatives that incentivise increased access to healthy food outlets in disadvantaged areas and better public transport infrastructure to increase accessibility. The impact of food environments on environmental sustainability should also be considered given poorer accessibility to food outlets increases reliance on cars (decreasing incidental physical activity) and creates barriers to accessing healthy food for those without cars^(1)^.

### Conclusion

Accessibility within a short walking distance to food outlets, particularly healthy food outlets and supermarkets, in Greater Melbourne is not equal. This inequality has remained despite increasing accessibility over time to these outlets. Understanding how people interact with food environments with different levels of accessibility to a range of food outlets will build on our understanding of how the food environment may contribute to unhealthy diets and needs to be considered in health promotion strategies and future research.

## Supporting information

Needham et al. supplementary material Needham et al. supplementary material

## Supplementary material

For supplementary material accompanying this paper visit https://doi.org/10.1017/S1368980024001344
