# Supplementary material for: Using spatial analysis to examine inequalities and temporal trends in food retail accessibility
Source: Public Health Nutr. 2024 Oct 24;27(1):e222. doi: 10.1017/S1368980024001344 (PMC11604324; doi:10.1017/S1368980024001344)
Supplement: Needham et al. supplementary material [file S1368980024001344sup001.docx]

**Supplementary Table 1: Food outlet descriptions and scores**

| Food outlet type | Description | Health Score |
| --- | --- | --- |
| Fruiterer & greengrocer | Mainly engaged in the sale of fresh fruit and vegetables; including wholesale stores with direct to public sales | 10 |
| Fish shop | Mainly engaged in the sale of fresh seafood; including wholesale stores with direct to public sales and takeaway stores also providing a range of fresh seafood. | 9 |
| Poultry shop | Mainly engaged in the sale of fresh poultry; including wholesale stores and with direct to public sales. | 9 |
| Butchery | Mainly engaged in the sale of fresh meat; including wholesale stores with direct to public sales. | 9 |
| Major Supermarket | Mainly engaged in the sale of groceries (fresh foods, canned and packaged foods, dry goods) of non-specialised (conventional) food lines. May contain a butcher or baker. Usually have 5 or more checkouts and a floor area over 1000 square meters. I.e. Woolworths, Coles, BI-LO, Franklins (no frills), ALDI | 5 |
| Minor Supermarket | Mainly engaged in the sale of groceries (fresh foods, canned and packaged food, dry goods) of non-specialised (conventional) food lines. Usually have 4 or fewer checkouts and a floor area under 1000 square meters. E.g. Independent grocer or supermarket. | 5 |
| Specialty food stores – core foods | Mainly engaged in the sale of a limited line of specialised food such as a particular gourmet food that can be defined under core food. | 5 |
| Restaurant/café – franchise | E.g. franchise restaurants and cafes; mainly engaged in the preparation and sale of meals/snacks for consumption on the premises; table service provided; may sell alcohol with food; may provide takeaway services. | 0 |
| Restaurant/café – local independent | e.g. restaurant in a golf club, culture-based restaurant/café which is not a take-away such as Mexican, Thai, Chinese etc. ; mainly engaged in the preparation and sale of meals/snacks for consumption on the premises; table service provided; may also sell alcohol with food, may provide takeaway services. | 0 |
| Sandwich shop | Mainly engaged in the preparation of filled bread products like sandwiches or rolls. | 5 |
| Salad/sushi bar | Mainly engaged in the preparation of salads and sushi. | 5 |
| Delicatessen | Mainly engaged in the sale of specialty packaged or fresh products such as cured meats and sausage, pickled vegetables, dips, bread and olives; may also provide dine in meals. | 0 |
| Bakery | Mainly oriented towards bread, biscuits, pastries or other flour products with or without packaging. | 0 |
| General store | Mainly engaged in the sale of a limited line of groceries generally includes milk, bread and canned and packaged foods. | -5 |
| Specialty food store – extra foods | Mainly engaged in the sale of foods such as ice-creams, donuts, waffles, cakes etc. than can be defined under extra food. | -8 |
| Pub | e.g. pub within a bowing park, pub inside a private gambling club; food primarily engaged in selling alcoholic beverages where consumers can order and consume the alcoholic drinks in premises; can also be part of park or private club. | -5 |
| Take-away local independent | E.g. kebab, fish & chips, burger, chicken shops, local pizza, mainly engaged in the preparation and sale of meals/snacks that are ready for immediate consumption; table service not provided; meals can be eaten on site; taken away or delivered; shop is not a franchise. | -8 |
| Take-away franchise store | E.g. McDonalds, KFC, Subway; mainly engaged in the preparation and sale of meals (excludes donuts, drinks, ice-cream etc.)/snacks that area ready for immediate consumption; table service not provided; meal can be eaten on site, taken away or delivered; the food shop is a franchise/chain store with food being sold in specialised packaging. | -10 |

Source: adapted from **Moayyed et al. 2017^(22)^**

[**https://doi.org/10.1111/1747-0080.12286**](https://doi.org/10.1111/1747-0080.12286)

**Supplementary Table 2. Food outlet classifications**

| Food outlets grouped by ‘type’ | Food outlets grouped by ‘healthiness’ score |
| --- | --- |
| 1. Supermarkets: Minor and Major Supermarkets | 1. Healthy (FES range: +5 to +10): Supermarkets, Fruit and greengrocer, Butcher, Fish, Poultry shop, Salad/Sandwich/Sushi bar. |
| 2. Fresh Produce: Fruit and greengrocer, Butcher, Fish, Poultry shop |
| 3. Eating Out: Cafes and Restaurants (Independent and Franchise), and Pubs | 1. Less Healthy (FES range: -4 to +4): Cafes and Restaurants (Independent and Franchise), Bakers, Delis. |
| 4. Small Goods: Bakers, Delis, Sandwich and Sushi |
| 5. Fast-food: Takeaway Franchise | 1. Unhealthy (FES range: -10 to -5): Fast-food, Takeaway independent, Pubs, General stores and Specialty extra. |
| 6. Takeaways: Takeaway Independent |
| 7. Discretionary Foods*: General stores and Specialty extra |

FES: Food Environment Score reflects the perceived healthiness value allocated to particular food outlet types. Range: -10 to +10

*Discretionary Foods are described in the Australian Dietary Guidelines as foods and drinks “not necessary for a healthy diet and are too high in saturated fat and/or added sugars, added salt or alcohol or low in fibre”(79)

Source: Needham et al(35)

<https://pubmed.ncbi.nlm.nih.gov/32092853/>

**Supplementary Table 3. Local government area classification by distance from the Central Business District in Melbourne, Australia**

| Local government area classification by distance to CBD | | |
| --- | --- | --- |
| LGA-Ring classification | Distance from CBD | Local Government Area ^#^ |
| Inner | <15km | Glen Eira, Maribyrnong, Melbourne (excluding CBD), Port Phillip, Stonington, Yarra |
| Middle | 15- 25km | Banyule, Bayside, Boroondara, Brimbank, Darebin, Hobsons Bay, Kingston, Manningham, Monash, Moonee Valley, Moreland, Whitehorse |
| Outer | 25-55km | Frankston, Dandenong, Knox, Maroondah, Mornington Peninsula, Nillumbik, Yarra Ranges |
| Growth Areas* | 30-70km | Casey, Cardinia, Hume, Melton, Whittlesea, Wyndham |

Acronym: CBD: Central Business District, Growth Areas: Victorian Growth Areas are local government areas designated by state government to house new population growth

Source: (35)

<https://pubmed.ncbi.nlm.nih.gov/32092853/>

**Supplementary Table 4. Material - Geo-FERN Reporting Checklist**

| **Geo-FERN (Geographic Information System Food Environment ReportiNg) Checklist** | | |
| --- | --- | --- |
| **INSTRUCTIONS** | | |
| For each reporting item, insert a tick or cross in the shaded box to indicate whether the item has been reported, or insert ‘N/A’ if not applicable. Shading indicates whether items are essential or desirable. Reporting items can be included in supplementary materials if word limits are tight and if allowed by the publisher. | | |
| 1. **FOOD OUTLET DATA** | **Essential** | **Desirable** |
| Name of the data creator (e.g. ‘Yellow Pages’, ‘Dunn & Bradstreet’ etc.). | **✓** |  |
| Collection and/or publication year of the data (include both if known). | **✓** |  |
| Title of the dataset. | **N/A** |  |
| Digital identifier of the dataset (e.g. a web address or DOI). |  | **N/A** |
| Publisher of the dataset. |  | **N/A** |
| Scope of the dataset (i.e. the geographic coverage of the dataset e.g. ‘national’ or ‘regional’ and the range of businesses included in the dataset, including any notable exclusions). |  | **✓** |
| Identification of the data fields used in analyses. |  | **✓** |
| Original purpose of the data (e.g. food hygiene regulation enforcement or commercial business data). |  | **✓** |
| Methods used by the data creator to collect the data/compile the dataset (e.g. audits conducted by data creator). |  | **✓** |
| Prevalence of missing data (e.g. number of entries with incomplete address information). |  | Method reported but data not provided |
| Methods for handling missing data (e.g. case-wise deletion, or use of secondary sources to impute missing data). |  | **✓** |
| Information on the accuracy of the data e.g. via reference to one or more validation studies or acknowledgement that data accuracy is unknown. |  | **✓** |
| 1. **EXTRACTING FOOD OUTLETS** | **Essential** | **Desirable** |
| Description of methods used to extract food outlets of interest from dataset (e.g. search for specific proprietary classifications or store names). | **✓** |  |
| If outlets were extracted using search terms (e.g. proprietary classifications or store names):   - An exhaustive list of search terms (where proprietary classifications are used, it should be made explicitly clear that the classifications listed are those of the data provider). | **✓** |  |
| If outlets were extracted based on proprietary classifications:   - A copy of the proprietary classification scheme, optionally including exemplary outlets falling within each classification; OR, - A discussion of any notable categories excluded from analyses (e.g. pubs, pharmacies, mobile food vendors etc.). |  | **✓** |
| 1. **DEFINING FOOD OUTLET CONSTRUCTS** | **Essential** | **Desirable** |
| Construct name(s) (e.g. ‘supermarkets’, ‘healthy outlets’, ‘convenience stores’ etc.). | **✓** |  |
| Description of the methods used to group outlets into constructs, including at least one of:   - An *exhaustive* list of any list-based criteria used to define each construct. This could include e.g. proprietary classifications making up each construct, or a list of store names making up each construct. Where proprietary classifications are used, it should be made explicitly clear that the classifications listed are those of the data provider. - Any objective criteria e.g. floor space, number of tills etc. used to define constructs. - **Citation of any previously published categorisation schemes that have been applied to the data and description of the methods used to apply the scheme.** - Description of any other methods used (note methods based on subjective criteria are discouraged). | **✓** |  |
| Examples of outlets falling within each construct such that the scope of each construct can be more readily interpreted. For example, if the construct ‘fast food outlet’ includes ‘traditional’ burger and fried chicken outlets, and also coffee shops and sandwich shops then well-known chains falling within each such sub-type could be listed. | **N/A** |  |
| Identification of any additional data sources used to group outlets into constructs e.g. use of Google Street View, business directories etc. |  | **✓** |
| Description of how any additional data sources were linked to the food outlet data (e.g. by matching store names and/or addresses). |  | **✓** |
| Where proprietary classifications are used to define constructs, a copy of the entire proprietary classification scheme. |  | **N/A** |
| 1. **GEOCODING METHODS** | **Essential** | **Desirable** |
| Acknowledgement of whether any data has been geocoded. | **✓** |  |
| The address model used (e.g. areal unit, street segment, land parcel, address point). | **✓** |  |
| The match rate achieved. |  |  |
| The environmental context, including details on how this was defined e.g. the study area was urban/rural, defined based on population density. | **✓** |  |
| Geocoding software used, including the version number. | **N/A** |  |
| The source of geocoding reference data (e.g. street line segment data), including publication date. |  | **✓** |
| 1. **ACCESS METRICS** | **Essential** | **Desirable** |
| Definition of the conceptual environment being measured e.g. home, school, work etc. | **✓** |  |
| **Intensity Metrics** | | |
| If areal zoning system used:   - The type of areal zoning system (e.g. government districts, census tracts etc.) - The source of boundary data, including the publication date or other version identifier. | **✓** |  |
| If buffer zoning system used:   - The buffer size. - The type of distance measure (e.g. Euclidian or network). | **N/A** |  |
| The units of the intensity metric(s) (e.g. count per unit area, as measured in meters) or formula indicating how they were calculated. | **N/A** |  |
| If network data was used (i.e. to calculate network distances):   - The source and publication date of network data. - The types of road/path included. | **✓** |  |
| Rationale for the choice of zone type (e.g. areal vs buffer) and/or size as applicable. |  | **✓** |
| **Proximity Metrics** | | |
| The type of distance measure (Euclidian vs network). | **✓** |  |
| If network data was used (i.e. to calculate network distances):   - The source and publication date of network data. - The types of road/path included. | **✓** |  |
| **Gravity Metrics** | | |
| The zone radius. | **N/A** |  |
| The decay coefficient. | **N/A** |  |
| 1. **UNKNOWN DETAILS** | **Essential** | **Desirable** |
| Any items noted as essential, but that are unknown should be highlighted as a limitation. | **Limitations noted regarding cost impedance in the network** |  |

**Supplementary Table 5.** **Spatial accessibility to food retail outlets in Greater Melbourne 2008-2016**

|  | **Year** | **Healthy** | **Less Healthy** | **Unhealthy** | **Discretionary Foods** | **Eating Out** | **Fast-food** | **Fresh Produce** | **Small Goods** | **Supermarkets** | **Takeaways** |
| --- | --- | --- | --- | --- | --- | --- | --- | --- | --- | --- | --- |
| 1KM | 2008 | 48.8% | 61.1% | 70.7% | 49.5% | 55.3% | 23.6% | 37.4% | 46.9% | 34.8% | 60.7% |
|  | 2012 | 50.3% | 61.8% | 70.6% | 46.9% | 56.8% | 28.9% | 36.9% | 46.4% | 41.9% | 61.0% |
|  | 2014 | 55.6% | 63.0% | 73.8% | 51.8% | 58.8% | 32.4% | 40.1% | 46.7% | 45.2% | 62.8% |
|  | 2016 | 53.0% | 63.6% | 74.0% | 52.8% | 59.5% | 34.1% | 38.1% | 46.8% | 45.8% | 63.5% |
| **Change** |  | **4.2%** | **2.5%** | **3.3%** | **3.3%** | **4.2%** | **10.5%** | **0.7%** | **-0.2%** | **11.0%** | **2.8%** |
|  | **Year** | **Healthy** | **Less Healthy** | **Unhealthy** | **Discretionary Foods** | **Eating Out** | **Fast-food** | **Fresh Produce** | **Small Goods** | **Supermarkets** | **Takeaways** |
| 3.2KM | 2008 | 89.6% | 92.3% | 94.5% | 89.2% | 92.6% | 84.5% | 87.4% | 90.1% | 85.0% | 92.1% |
|  | 2012 | 91.9% | 92.3% | 94.8% | 90.6% | 92.8% | 87.8% | 90.6% | 90.1% | 89.6% | 91.5% |
|  | 2014 | 94.3% | 93.4% | 95.6% | 91.5% | 93.5% | 88.8% | 89.8% | 91.2% | 93.6% | 93.5% |
|  | 2016 | 93.5% | 93.9% | 95.7% | 92.0% | 94.1% | 89.4% | 88.1% | 91.3% | 92.8% | 93.6% |
| **Change** |  | **3.9%** | **1.6%** | **1.2%** | **2.8%** | **1.5%** | **4.9%** | **0.7%** | **1.2%** | **7.8%** | **1.5%** |

**Supplementary Table 6a.** **Sidak adjusted pairwise comparisons between LGA-Rings within year or between years within LGA-Rings for proportion of the population with access to different type of food outlets within 1km service area classified by healthiness.**

Sidak adjusted pairwise comparisons are obtained under linear mixed including year (2008, 2012, 2014, 2016), LGA-Ring (Inner, Middle, Outer, Growth Area) and the interaction LGA-Ring by year as fixed effects and LGA as random effect; only comparisons where the main effect was significant are reported.

*Significance pairwise comparisons: Bold <0.01**.** Underlined p<0.05.

**Supplementary Table 6b.** **Sidak adjusted pairwise comparisons between LGA-Rings within year or between years within LGA-Rings for proportion of the population with access to different type of food outlets within 1km service area classified by food outlet type.**

Sidak adjusted pairwise comparisons are obtained under linear mixed including year (2008, 2012, 2014, 2016), LGA-Ring (Inner, Middle, Outer, Growth Area) and the interaction LGA-Ring by year as fixed effects and LGA as random effect; only comparisons where the main effect was significant are reported.

*Significance pairwise comparisons: Bold <0.01**.** Underlined p<0.05.

**Supplementary Table 7. Sidak adjusted pairwise comparisons between LGA-Rings or within year for 1km service area measures where interactions are non-significant and main effects are significant**

|  | **Discretionary** | **Small Goods** | **Takeaways** |
| --- | --- | --- | --- |
| LGA comparison |
| Middle vs Inner | **-0.25(-0.42, -0.09)** | **-0.26(-0.4, -0.12)** | -0.16(-0.35, 0.02) |
| Outer vs Inner | **-0.49(-0.67, -0.31)** | **-0.55(-0.7, -0.39)** | **-0.5(-0.71, -0.29)** |
| Growth vs Inner | **-0.57(-0.76, -0.38)** | **-0.63(-0.79, -0.47)** | **-0.61(-0.82, -0.4)** |
| Outer vs Middle | **-0.24(-0.4, -0.08)** | **-0.29(-0.42, -0.15)** | **-0.34(-0.51, -0.16)** |
| Growth vs Middle | **-0.32(-0.48, -0.15)** | **-0.37(-0.51, -0.23)** | **-0.45(-0.63, -0.26)** |
| Growth vs Outer | -0.08(-0.26, 0.1) | -0.08(-0.24, 0.07) | -0.11(-0.32, 0.1) |
| Year comparison |
| 2012 vs 2008 | **-** | **-** | 0.01(-0.01, 0.03) |
| 2014 vs 2008 | **-** | **-** | **0.03(0.01, 0.06)** |
| 2016 vs 2008 | **-** | **-** | **0.04(0.02, 0.07)** |
| 2014 vs 2012 | **-** | **-** | 0.02(0, 0.05) |
| 2016 vs 2012 | **-** | **-** | **0.03(0.01, 0.06)** |
| 2016 vs 2014 | **-** | **-** | 0.01(-0.01, 0.04) |

Sidak adjusted pairwise comparisons are obtained under linear mixed including year (2008, 2012, 2014, 2016), LGA-Ring (Inner, Middle, Outer, Growth Area) and the interaction LGA-Ring by year as fixed effects and LGA as random effect; only comparisons where the main effect was significant are reported.

Bold: P≤0.05

**Supplementary Table 8. Sum of local governments where most of (≥ 80%) the population live within 1km of food retail outlet types classified by healthiness and food outlet type.**

**Supplementary Table 9. Mean proportion of the population within local government areas classified by distance from the central business district and identified growth area within 3.2km of food retail outlets between 2008 and 2016.**

| **Local Governments classified by Distance from CBD and identified Growth Area** | | | | | | | | | | | | | | | | | | | |
| --- | --- | --- | --- | --- | --- | --- | --- | --- | --- | --- | --- | --- | --- | --- | --- | --- | --- | --- | --- |
|  | **Inner** | | | | **Middle** | | | | **Outer** | | | | **Growth** | | | | **Model** | | |
|  | **2008** | **2012** | **2014** | **2016** | **2008** | **2012** | **2014** | **2016** | **2008** | **2012** | **2014** | **2016** | **2008** | **2012** | **2014** | **2016** | **Year effect** | **LGA Ring effect** | **Interaction** |
| **Measure % 3.2km** | **Proportion of the population within 3.2km (%, 95% CI)** | | | | | | | | | | | | | | | |  | **P value** |  |
| **Classified by healthiness** | | | | | | | | | | | | | | | | | | | |
| **Healthy** | **100** | **100** | **100** | **100** | **100** | **100** | **100** | **100** | **80** | **80** | **86** | **84** | **68** | **82** | **87** | **85** | **1.0000** | **<0.0001** | **<0.0001** |
| **95% CI** | **89, 111** | **89, 111** | **89, 111** | **89, 111** | **92, 107** | **92, 107** | **92, 108** | **92, 108** | **69, 90** | **70, 90** | **75, 96** | **74, 94** | **57, 79** | **71, 93** | **76, 98** | **74, 96** |  |  |  |
| **Less Healthy** | **100** | **100** | **100** | **100** | **100** | **100** | **100** | **100** | **85** | **82** | **83** | **85** | **77** | **80** | **85** | **86** | **1.0000** | **0.0022** | **0.0001** |
| **95% CI** | **89, 111** | **89, 111** | **89, 111** | **89, 111** | **92, 108** | **92, 108** | **92, 108** | **92, 108** | **74, 95** | **72, 93** | **73, 94** | **75, 95** | **66, 88** | **69, 91** | **74, 96** | **75, 98** |  |  |  |
| **Unhealthy** | **100** | **100** | **100** | **100** | **100** | **100** | **100** | **100** | **87** | **87** | **87** | **88** | **85** | **88** | **91** | **92** | **1.0000** | **0.0037** | **0.0002** |
| **95% CI** | **92, 108** | **92, 108** | **92, 108** | **92, 108** | **94, 105** | **94, 106** | **94, 106** | **94, 106** | **80, 95** | **79, 95** | **80, 95** | **80, 95** | **76, 93** | **80, 96** | **83, 99** | **84, 100** |  |  |  |
| **Classified by food outlet type** | | | | | | | | | | | | | | | | | | | |
| **Discretionary** | **100** | **100** | **100** | **100** | **99** | **99** | **99** | **99** | **81** | **79** | **80** | **80** | **67** | **75** | **81** | **82** | **1.0000** | **0.0004** | **<0.0001** |
| **95% CI** | **86, 114** | **86, 114** | **86, 114** | **86, 114** | **89, 109** | **89, 109** | **89, 109** | **90, 109** | **68, 94** | **67, 92** | **67, 92** | **67, 93** | **53, 80** | **61, 88** | **67, 94** | **69, 96** |  |  |  |
| **Eating out** | **100** | **100** | **100** | **100** | **100** | **100** | **100** | **100** | **84** | **84** | **85** | **86** | **79** | **81** | **85** | **86** | **1.0000** | **0.0005** | **0.0014** |
| **95% CI** | **90, 110** | **90, 110** | **90, 110** | **90, 110** | **93, 107** | **93, 107** | **93, 107** | **93, 107** | **75, 93** | **75, 93** | **76, 93** | **77, 95** | **70, 89** | **72, 91** | **76, 95** | **77, 96** |  |  |  |
| **Fast-food** | **100** | **100** | **100** | **100** | **93** | **96** | **98** | **97** | **69** | **74** | **75** | **75** | **65** | **73** | **76** | **79** | **0.9997** | **<0.0001** | **0.0305** |
| **95% CI** | **88, 112** | **88, 112** | **88, 112** | **88, 112** | **85, 102** | **87, 104** | **89, 106** | **89, 106** | **58, 80** | **63, 85** | **64, 86** | **64, 87** | **53, 77** | **61, 85** | **64, 88** | **67, 91** |  |  |  |
| **Fresh produce** | **100** | **100** | **100** | **100** | **97** | **100** | **100** | **100** | **78** | **78** | **77** | **77** | **63** | **79** | **78** | **72** | **1.0000** | **<0.0001** | **0.0001** |
| **95% CI** | **87, 113** | **87, 113** | **87, 113** | **87, 113** | **88, 106** | **90, 109** | **90, 109** | **90, 109** | **66, 90** | **66, 90** | **65, 89** | **65, 89** | **50, 77** | **65, 92** | **65, 91** | **59, 86** |  |  |  |
| **Small goods** | **100** | **100** | **100** | **100** | **100** | **100** | **100** | **100** | **80** | **79** | **80** | **80** | **73** | **75** | **81** | **82** | **1.0000** | **0.0007** | **<0.0001** |
| **95% CI** | **87, 113** | **87, 113** | **87, 113** | **87, 113** | **91, 109** | **91, 109** | **91, 109** | **91, 109** | **69, 92** | **68, 91** | **68, 91** | **68, 91** | **60, 85** | **63, 88** | **68, 93** | **69, 94** |  |  |  |
| **Supermarkets** | **100** | **100** | **100** | **100** | **97** | **100** | **100** | **100** | **78** | **79** | **84** | **84** | **55** | **74** | **86** | **83** | **1.0000** | **<0.0001** | **<0.0001** |
| **95% CI** | **89, 111** | **89, 111** | **89, 111** | **89, 111** | **89, 105** | **92, 107** | **92, 107** | **92, 108** | **68, 88** | **69, 89** | **74, 95** | **74, 94** | **44, 66** | **63, 85** | **75, 97** | **72, 94** |  |  |  |
| **Takeaways** | **100** | **100** | **100** | **100** | **100** | **100** | **100** | **100** | **82** | **82** | **82** | **82** | **80** | **80** | **87** | **88** | **1.0000** | **0.0037** | **<0.0001** |
| **95% CI** | **89, 111** | **89, 111** | **89, 111** | **89, 111** | **92, 107** | **92, 108** | **92, 108** | **92, 108** | **71, 92** | **72, 92** | **72, 93** | **72, 93** | **69, 92** | **69, 91** | **76, 98** | **77, 99** |  |  |  |

Mean estimates and 95% confidence intervals obtained under linear mixed including year (2008, 2012, 2014, 2016), LGA-Ring (Inner, Middle, Outer, Growth Area) and the interaction LGA-Ring by year as fixed effects and LGA as random effect; significance of Interaction and test for main effects from the model is also reported.

Bold: P≤0.05

**Supplementary Table 10. Sidak adjusted pairwise comparisons between LGA-Rings within year or between years within LGA-Rings for linear mixed model for 3.2km service area for food retail outlets classified by healthiness.**

Sidak adjusted pairwise comparisons are obtained under linear mixed including year (2008, 2012, 2014, 2016), LGA-Ring (Inner, Middle, Outer, Growth Area) and the interaction LGA-Ring by year as fixed effects and LGA as random effect; only comparisons where the interaction was significant are reported.

Bold: P≤0.01 *≤0.05

**Supplementary Table 11. Sidak adjusted pairwise comparisons between LGA-Rings within year or between years within LGA-Rings for linear mixed model for 3.2km service area for food retail outlets classified by type.**

Sidak adjusted pairwise comparisons are obtained under linear mixed including year (2008, 2012, 2014, 2016), LGA-Ring (Inner, Middle, Outer, Growth Area) and the interaction LGA-Ring by year as fixed effects and LGA as random effect; only comparisons where the interaction was significant are reported.

Bold: P≤0.05

**Supplementary Table 12. Sidak adjusted pairwise comparisons between LGA-Rings or within year for linear mixed model for 3.2km service area where main effects are significant**

|  | **Fast-food** |
| --- | --- |
| **LGA comparison** | Contrast (95%CI) |
| Middle vs Inner | -0.04(-0.23, 0.15) |
| Outer vs Inner | **-0.26(-0.48, -0.05)** |
| Growth vs Inner | **-0.26(-0.48, -0.05)** |
| Outer vs Middle | **-0.22(-0.41, -0.04)** |
| Growth vs Middle | -0.23(-0.42, -0.04)* |
| Growth vs Outer | 0(-0.21, 0.21) |

Sidak adjusted pairwise comparisons are obtained under linear mixed including year (2008, 2012, 2014, 2016), LGA-Ring (Inner, Middle, Outer, Growth Area) and the interaction LGA-Ring by year as fixed effects and LGA as random effect; only comparisons where the main effect was significant are reported.

Bold: P≤0.05
